# Assessment of thyroid hormone profiles and derived ratios for predicting thyroid nodule malignancy using the Bethesda system: thyroid hormone ratios in malignancy risk stratification of thyroid nodules: a cross-sectional study

**DOI:** 10.1590/1516-3180.2025.3317.08062026

**Published:** 2026-08-14

**Authors:** İrem Şenoymak, Mustafa Can Şenoymak, Memet Taşkın Egici, İrem Kahveci, İsra Oğuz, Adnan Gökçel

**Affiliations:** I MD. Physician, Department of Family Medicine, University of Health Sciences Şehit Prof. Dr. İlhan Varank Training and Research Hospital, Istanbul, Türkiye.; II MD. Physician, Department of Endocrinology and Metabolism, University of Health Sciences Sultan II. Abdulhamid Han Training and Research Hospital, Istanbul, Türkiye.; III MD. Professor, Department of Family Medicine, University of Health Sciences Haydarpaşa Numune Training and Research Hospital, Istanbul, Türkiye.; IV MD. Physician, Department of Family Medicine, University of Health Sciences Haydarpaşa Numune Training and Research Hospital, Istanbul, Türkiye.; V MD. Physician, Department of Endocrinology and Metabolism, University of Health Sciences Haydarpaşa Numune Training and Research Hospital, Istanbul, Türkiye.; VI MD. Professor, Department of Endocrinology and Metabolism, University of Health Sciences Haydarpaşa Numune Training and Research Hospital, Istanbul, Türkiye.

**Keywords:** Thyroid Nodule, Biopsy, Thyroid Function Tests, Thyroid Cancer, Cytodiagnosis, Thyrotropin, Thyroid Neoplasms, Fine Needle Aspiration Biopsy, Hormonal Ratio Biomarkers, Preoperative Risk Assessment

## Abstract

**BACKGROUND:**

Thyroid nodules are common, and although most are benign, a proportion may be malignant. Fine-needle aspiration biopsy (FNAB), interpreted using the Bethesda System, is the standard diagnostic method. However, in euthyroid patients, the predictive value of thyroid function tests and their derived ratios in relation to Bethesda categories remains unclear.

**OBJECTIVES:**

To evaluate thyroid function parameters and their association with Bethesda classification in euthyroid patients with thyroid nodules, and to identify potential predictors of malignancy.

**DESIGN AND SETTING:**

This retrospective cross-sectional study was conducted at the University of Health Sciences Haydarpaşa Numune Training and Research Hospital and included a convenience sample of eligible euthyroid patients who underwent FNAB between January 2020 and December 2023.

**METHODS:**

A total of 828 euthyroid patients were included. Demographic data, pre-biopsy TSH, fT3, fT4, and derived ratios (TSH/T4, TSH/T3, and T3/T4) were analyzed for correlations with Bethesda categories and compared between the benign and malignant groups.

**RESULTS:**

The mean age was 52.85 ± 12.53 years, and 80.8% of participants were female. Benign cytology was found in 54.6% of cases, and malignant cytology was found in 2.8%. Cases with malignant cytology were younger (p = 0.003) and more frequently male (p = 0.021). No significant differences were observed in hormone levels or ratios between the benign and malignant groups. However, TSH and the TSH/T4 ratio showed positive correlations with Bethesda category (p = 0.034 and p = 0.033, respectively).

**CONCLUSION:**

The TSH level and TSH/T4 ratio were correlated with Bethesda categories; however, thyroid function tests and derived ratios showed no diagnostic value for predicting malignancy in euthyroid nodules.

## INTRODUCTION

Thyroid nodules are common thyroid disorders frequently encountered in clinical practice and are highly prevalent in the general population. The prevalence of thyroid nodules varies depending on the studied population and the diagnostic method used, ranging from 2-6% by palpation, 19-35% by ultrasound, and 8-65% in autopsy studies.^1^

Most thyroid nodules are benign; however, approximately 5-15% are malignant.^2^ Accurate evaluation and management of nodules are crucial for both clinical decision-making and public health. Therefore, the primary goal in the management of patients with thyroid nodules is to assess the risk of malignancy. A definitive diagnosis of malignancy or benign disease can be established only through histopathological examination. Thyroid fine-needle aspiration biopsy (FNAB) should be performed on nodules with suspicious malignant features based on history, physical examination, laboratory findings, and ultrasonographic characteristics.^3^ The Bethesda System for Reporting Thyroid Cytopathology was developed to standardize FNAB cytology and provide clinicians with guidance on malignancy risk estimation and patient management. According to this system, FNAB results are categorized as follows: Bethesda 1 - non-diagnostic, Bethesda 2 - benign, Bethesda 3 - atypia of undetermined significance, Bethesda 4 - follicular neoplasm, Bethesda 5 - suspicious for malignancy, and Bethesda 6 - malignant.^4^ Bethesda 3 and 4 are collectively referred to as indeterminate cytology.

Elevated serum thyroid-stimulating hormone (TSH) levels have been suggested to increase the risk of thyroid malignancy in patients with nodular thyroid disease. Several studies have demonstrated that even within the normal range, higher TSH values are associated with a greater frequency and more advanced stages of thyroid cancer.^5^ ^,^ ^6^ Free triiodothyronine (T3) and free thyroxine (T4) levels have also been investigated in the literature, with conflicting results. A meta-analysis indicated that higher T3 levels were associated with a 14% reduction in thyroid cancer risk.^7^ However, other studies suggest that T3 and other thyroid hormones may influence cancer cell growth, proliferation, and metastasis.^8^ ^,^ ^9^ In addition, another study demonstrated that elevated free T4 and free T3 levels are associated with the development of thyroid cancer in both sexes.^10^

Although TSH levels are thought to be associated with malignancy in thyroid nodules, there are insufficient data on the relationship between TSH levels and preoperative cytopathological findings. Therefore, in this study, we aimed to investigate the correlations between Bethesda classification and TSH, free T3, free T4, the T3/T4 ratio, the TSH/T3 ratio, and the TSH/T4 ratio in euthyroid individuals. Our literature review indicates that studies comprehensively examining the relationship between the Bethesda classification and thyroid function parameters are limited. The objective of this study was to identify a readily applicable, non-invasive laboratory parameter with a potential cutoff value for predicting thyroid malignancy.

## OBJECTIVE

The primary objective of this study was to investigate potential associations between thyroid function parameters and the Bethesda classification in patients with euthyroid thyroid nodules. Specifically, this study aimed to determine whether serum levels of thyroid-stimulating hormone (TSH), free thyroxine (fT4), and free triiodothyronine (fT3), as well as their derived ratios (TSH/fT4, TSH/fT3, and fT3/fT4), had a statistically significant relationship with cytopathological findings obtained through fine-needle aspiration biopsy (FNAB). By comparing these parameters between patients classified as having benign (Bethesda category II) and malignant (Bethesda category VI) nodules, the study sought to identify accessible and cost-effective biochemical predictors that may aid pre-biopsy risk stratification and clinical decision-making in thyroid nodule management.

## METHODS

### Study design and population

This retrospective cross-sectional study was conducted with the approval of the University of Health Sciences Haydarpaşa Numune Training and Research Hospital Ethics Committee (approval no. HNEAH-KAEK 2024/2 24/110) and adhered to the principles outlined in the Declaration of Helsinki. A convenience sampling method was employed. All patients aged 18 years or older who underwent thyroid fine-needle aspiration biopsy (FNAB) between January 1, 2020, and December 31, 2023, at the University of Health Sciences Haydarpaşa Numune Training and Research Hospital, and whose cytopathological results were available in the hospital records, were included in the study. The final sample size was determined by the number of patients who met the inclusion and exclusion criteria within the specified study period. This sample was considered representative of the institution’s real-world patient population during this timeframe.

Given the retrospective design of the study and the availability of comprehensive electronic medical records, this approach enabled the inclusion of a broad, unselected patient population, thereby enhancing the external validity and generalizability of the findings. Moreover, the temporal scope of data collection allowed diagnostic and clinical patterns to be captured over a multi-year period, which may further strengthen the robustness of the observed associations.

### Data collection and definitions

Demographic variables (age and sex), cytopathological results of FNAB, and serum levels of thyroid-stimulating hormone (TSH), free thyroxine (fT4), and free triiodothyronine (fT3), measured within the three months preceding the biopsy, if available, were recorded. Derived ratios, including TSH/fT4, TSH/fT3, and fT3/fT4, were then calculated. The relationships between these parameters and Bethesda classification categories were analyzed. In addition, patients classified as benign (Bethesda category II) and malignant (Bethesda category VI) were compared with respect to these variables.

### Exclusion criteria

The study excluded individuals younger than 18 years of age and those with known primary thyroid diseases, including hypothyroidism, subacute thyroiditis, thyrotoxicosis, Hashimoto’s thyroiditis, or Graves’ disease; advanced-stage hepatic or renal disease; pituitary disorders; or treatment with antithyroid medications, levothyroxine sodium, or triiodothyronine. Additional exclusion criteria included the use of medications known to interfere with thyroid function, such as estrogen, androgen, lithium, or glucocorticoids; the presence of active infections or malignancies other than thyroid cancer; and incomplete or missing data.

### Statistical analysis

All statistical analyses were performed using IBM SPSS Statistics 21.0 (Armonk, New York: IBM Corp.) and MS Excel 2007. The normality of continuous variables was assessed both graphically and using the Shapiro-Wilk test. Descriptive statistics were presented as mean ± standard deviation (SD) and median (minimum-maximum) values.

To compare sex distribution between the benign and malignant groups, cross-tabulations were created, and the number (n), percentage (%), and Fisher exact test statistic were reported. The Mann-Whitney U test was used to compare age, TSH, free T4, free T3, TSH/T4, TSH/T3, and T3/T4 values between the benign and malignant groups. To identify potential risk factors, receiver operating characteristic (ROC) analysis was conducted for TSH, free T4, free T3, TSH/T4, TSH/T3, and T3/T4 values, with the benign-malignant classification as the reference. The ROC curve was plotted, and the area under the curve (AUC), together with its 95% confidence interval (95% CI) was determined. For correlation analysis between the Bethesda classification and TSH, free T4, free T3, TSH/T4, TSH/T3, and T3/T4 values, Spearman’s non-parametric correlation coefficient was calculated. Bethesda categories were treated as ordinal variables in the correlation analyses to assess monotonic trends across increasing malignancy risk; however, it is acknowledged that, clinically, the Bethesda classification represents a categorical and non-linear risk stratification system. A p value of < 0.05 was considered statistically significant.

## RESULTS

Data from 1,719 individuals were initially collected, and analyses were conducted on 828 individuals who met the inclusion criteria. Of these, 80.8% (n = 669) were female, and 19.2% (n = 159) were male. The mean age of the participants was 52.85 ± 12.53 years.

Regarding the Bethesda classification, 24.5% (n = 203) of cases were categorized as Bethesda 1, while 54.6% (n = 453) were categorized as Bethesda 2. Bethesda 3 accounted for 14.9% (n = 123), Bethesda 4 for 1.0% (n = 8), Bethesda 5 for 2.2% (n = 18), and Bethesda 6 for 2.8% (n = 23).

The mean TSH level was 1.74 ± 1.06, while the mean free T3 and free T4 levels were 3.06 ± 0.43 and 1.16 ± 0.18, respectively. Additionally, the mean TSH/T4, TSH/T3, and T3/T4 ratios were 1.55 ± 1.01, 0.56 ± 0.36, and 2.69 ± 0.51, respectively (Table 1). The mean age of individuals with benign cytology was 53.73 ± 12.05 years, whereas that of individuals with malignant cytology was 45.78 ± 12.56 years. A statistically significant difference in age was observed between the benign and malignant groups (z = 2.927, p = 0.003).

**Table 1.** Demographic and clinical characteristics of the participants

		Participants (n = 828)
Sex, n (%)		
*Male*		159 (19.2)
*Female*		669 (80.8)
Age (years)	*Mean* ± *SD*	52.85 ± 12.53
	*Median (min-max)*	53.0 (19-85)
Bethesda, n (%)		
*1*		203 (24.5)
*2*		453 (54.6)
*3*		123 (14.9)
*4*		8 (1)
*5*		18 (2.2)
*6*		23 (2.8)
TSH	*Mean* ± *SD*	1.74 ± 1.06
	*Median (min-max)*	1.42 (0.454-4.99)
T3	*Mean* ± *SD*	3.06 ± 0.43
	*Median (min-max)*	3.05 (2.08-4.33)
T4	*Mean* ± *SD*	1.16 ± 0.18
	*Median (min-max)*	1.15 (0.81-1.7)
TSH/T4	*Mean* ± *SD*	1.55 ± 1.01
	*Median (min-max)*	1.24 (0.30-5.8)
TSH/T3	*Mean* ± *SD*	0.56 ± 0.36
	*Median (min-max)*	0.46 (0.12-1.9)
T3/T4	*Mean* ± *SD*	2.69 ± 0.51
	*Median (min-max)*	2.66 (1.39-4.55)

TSH: thyroid-stimulating hormone; SD: standard deviation.

In the benign group, 81.5% (n = 369) were female and 18.5% (n = 84) were male, whereas in the malignant group, 60.9% (n = 14) were female and 39.1% (n = 9) were male. Sex distribution differed significantly between the benign and malignant groups (p = 0.021).

**Figure 1.**
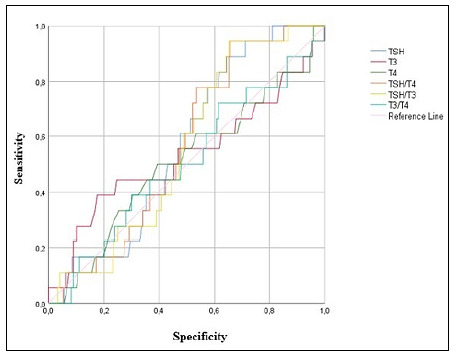
ROC analyses for TSH, T4, T3, TSH/T4, TSH/T3, and T3T4.

No statistically significant differences were found between the benign and malignant groups in terms of TSH, T3, T4, TSH/T4, TSH/T3, or T3/T4 values (p > 0.05) (Table 2). ROC analysis based on the benign versus malignant classification revealed that the area under the curve (AUC) values for TSH, T3, T4, TSH/T4, TSH/T3, and T3/T4 were not statistically significant (p > 0.05) (Table 3, Figure 1).

**Table 2.** Comparison of demographic and clinical characteristics between benign and malignant groups

	Benign (n = 453)	Malign (n = 23)		
	Mean ± SD Median (Min-Max)	Mean±SD Median (Min-Max)	z	p
Age	53.73 ± 12.05	45.78 ± 12.56	2.927	0.003
	54 (19-85)	44 (20-68)		
Sex, n (%)				
*Male*	84 (18.5)	9 (39.1)	-	0.021*
*Female*	369 (81.5)	14 (60.9)		
TSH	1.68 ± 1.06	1.82 ± 1.1	0.937	0.349
	1.37 (0.45-4.99)	1.54 (0.64-4.53)		
T3	3.04 ± 0.41	3.01 ± 0.56	0.441	0.659
	3.03 (2.11-4.19)	2.98 (2.09-4.24)		
T4	1.16 ± 0.18	1.14 ± 0.18	0.834	0.404
	1.16 (0.81-1.7)	1.1 (0.9-1.49)		
TSH/T4	1.49 ± 1.03	1.63 ± 1.01	1.081	0.279
	1.16 (0.3-5.8)	1.23 (0.56-3.91)		
TSH/T3	0.55 ± 0.37	0.59 ± 0.36	0.886	0.376
	0.43 (0.13-1.9)	0.47 (0.21-1.54)		
T3/T4	2.65 ± 0.51	2.62 ± 0.52	0.089	0.929
	2.63 (1.39-4.55)	2.6 (1.4-3.4)		

Z: Mann-Whitney U test; *Fisher exact test. TSH: thyroid-stimulating hormone; SD: standard deviation.

**Table 3.** Area under the curve (AUC) analysis for malignancy prediction

	AUC ± SE	95% CI	Cut.	p	Sensitivity (%)	Specificity (%)
TSH	0.558 ± 0.053	0.454-0.662	-	0.349	-	-
T3	0.531 ± 0.083	0.368-0.694	-	0.659	-	-
T4	0.552 ± 0.064	0.427-0.676	-	0.404	-	-
TSH/T4	0.567 ± 0.052	0.466-0.668	-	0.279	-	-
TSH/T3	0.562 ± 0.054	0.456-0.668	-	0.376	-	-
T3/T4	0.506 ± 0.071	0.366-0.646	-	0.929	-	-

AUC: area under the curve; SE: standard error; CI: confidence interval; TSH: thyroid-stimulating hormone.

**Figure 2.**
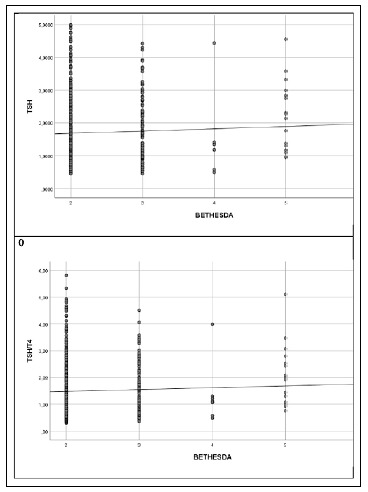
Correlation between Bethesda classification and TSH and the TSH/T4 ratio.

A weak but statistically significant positive correlation was found between Bethesda classification and both TSH (ρ = 0.085; p = 0.034) and TSH/T4 values (ρ = 0.086; p = 0.033). However, the strength of these correlations was very low, indicating limited practical or clinical significance despite statistical significance. As the Bethesda category increased (from 2 to 6), TSH and TSH/T4 values showed corresponding increases. No statistically significant correlations were observed between Bethesda classification and T4, T3, TSH/T3, or T3/T4 values (p > 0.05) (Table 4, Figure 2).

**Table 4.** Relationships between Bethesda classification and thyroid parameters

	Bethesda	
	ρ	p
TSH	0.085	0.034
T3	0.015	0.768
T4	−0.018	0.648
TSH/T4	0.086	0.033
TSH/T3	0.055	0.285
T3/T4	0.013	0.802

R: Spearman’s correlation coefficient; TSH: thyroid-stimulating hormone.

## DISCUSSION

In this study, we compared thyroid function test parameters between patients with benign and malignant cytology (Bethesda 6) and examined the correlations of TSH, free T3, T4, TSH/T4, TSH/T3, and T3/T4 ratios with Bethesda classification. In the current literature, few studies have investigated the relationship between Bethesda classification and thyroid function tests. In this context, our findings contribute to this underexamined area of research, with the additional strength of a relatively large participant cohort compared with those of existing studies.

Our study identified a statistically significant difference in mean age between the benign and malignant groups. The malignant group had a lower mean age than the benign group (45.78 ± 12.56 vs. 53.73 ± 12.05 years), suggesting that thyroid cancer may occur more frequently in younger age groups. This observation aligns with existing literature reporting a higher prevalence of malignant thyroid nodules in young adults, particularly in the 20-40-year age range, with increasing incidence rates.^11^ A cohort study further supports this pattern, demonstrating that while thyroid nodule prevalence increases with age, malignancy risk shows an inverse relationship.^12^ These findings underscore the clinical importance of thorough evaluation for malignancy in younger patients presenting with thyroid nodules.

Our analysis of sex distribution revealed a female predominance in the benign group (81.5%), while males had significantly higher representation in the malignant group (39.1%, p = 0.021). This finding suggests that male sex may be a potential risk factor for thyroid nodule malignancy. The literature presents conflicting evidence regarding sex-specific thyroid cancer risk. Although studies report a higher prevalence of nodules in women, the association between sex and malignancy risk remains inconsistent. Some studies indicate higher rates of differentiated thyroid cancers in females, potentially due to the proliferative effects of sex hormones (estrogen and progesterone) on thyroid cells.^13^ ^,^ ^14^ ^,^ ^15^ A recent cohort study found that female sex was specifically associated with increased papillary thyroid carcinoma risk, while other histological subtypes showed comparable incidence between sexes.^16^ However, most reports suggest that although male thyroid cancers are less common, they tend to demonstrate more aggressive behavior and are often diagnosed at advanced stages.^13^ ^,^ ^14^ Our results align with these observations: while thyroid nodules were more prevalent in women, male sex emerged as a potential malignancy risk factor.

Recent research has revealed that TSH not only regulates thyroid hormone synthesis but may also influence the development and progression of thyroid cancer. As a major growth factor for thyroid cells, TSH activates signaling pathways known to contribute to tumorigenesis.^17^ Differentiated thyroid cancer cells express TSH receptors, and TSH stimulation may enhance proliferation through increased expression of thyroid-specific proteins.^4^ However, the precise mechanisms remain unclear because of limited clinical research. Although multiple meta-analyses have associated elevated TSH levels with increased thyroid cancer risk,^5^ ^,^ ^18^ the evidence remains inconsistent. Boelaert et al.^19^ demonstrated that euthyroid patients with TSH levels of 1.0-1.7 mU/L had a higher malignancy risk than those with TSH < 0.4 mU/L, with a significantly increased risk at TSH > 1.8 mU/L. Baser et al.^20^ identified ≥ 1.24 mIU/mL as a potential TSH cutoff for malignancy prediction. However, a meta-analysis conducted in 2012 that evaluated surgically treated patients with nodular goiter found no significant association between TSH levels and thyroid cancer incidence.^21^ Another meta-analysis revealed that elevated TSH levels were associated with increased malignancy risk when compared with benign thyroid tumors. However, in the limited number of studies that used healthy individuals as controls, no statistically significant difference in malignancy risk was observed in relation to TSH levels.^7^ These discrepancies may reflect methodological differences across studies. In our study, although we found no significant difference in TSH levels between the benign and malignant groups (p > 0.05), correlation analysis revealed a weak but statistically significant positive association between Bethesda classification and both TSH and TSH/T4 ratios. This aligns with literature reporting progressively higher TSH levels from Bethesda 2 to 4 categories.^20^ ^,^ ^22^ Consequently, some researchers propose that elevated TSH levels could function as a supplementary biomarker in cases with suspicious or malignant cytology, although their definitive prognostic significance requires validation through larger-scale studies.^20^ However, the very weak correlation coefficients observed in our study suggest that these associations should be interpreted with caution and are unlikely to be of meaningful clinical utility on their own.

In addition to TSH, recent studies have highlighted the potential direct effects of thyroid hormones on thyroid cell proliferation. The T3 receptor has been identified as a regulator of thyroid fibroblast growth factor expression, and murine studies demonstrate that mutated T3 receptors may promote thyroid carcinogenesis by upregulating the phosphatidylinositol 3-kinase signaling pathway.^23^ Meta-analyses suggest that elevated free T3 levels are associated with reduced thyroid cancer risk, while free T4 levels show no significant correlation.^7^ Another study investigating the relationship between thyroid function and differentiated thyroid carcinomas found that lower free T3 and T4 concentrations within normal ranges independently predicted increased cancer risk, regardless of sex or nodule type.^24^ However, other studies report paradoxical findings. For instance, Kim et al.^10^ associated higher free T3 and T4 levels with incident thyroid cancer, whereas large-scale studies detected no significant relationship between free thyroid hormones and malignancy risk. In our cohort, free T3 and T4 levels showed no statistically significant association with malignancy when comparing the benign and malignant groups. However, we identified a potential link between the TSH/T4 ratio and Bethesda classification, warranting further investigation.

This study has several notable limitations that should be considered when interpreting the results. The primary limitation is the absence of histopathological confirmation for cytological findings, as fine-needle aspiration biopsy carries inherent risks of both false-positive and false-negative results, as acknowledged in current guidelines.^4^ In addition, the limited sample size of the malignant group may have reduced the statistical power of our analyses. This is particularly relevant for ROC and correlation analyses, where the small number of malignant cases (n = 23) increases the risk of type 2 error and may weaken the reliability of predictive performance estimates. Furthermore, all laboratory markers were measured only once within three months before biopsy, which may not capture hormonal fluctuations or adequately reflect patients’ long-term thyroid status. Taken together, these limitations should be considered when interpreting the observed associations. Importantly, the absence of significant findings in ROC analysis indicates that these biochemical parameters lack sufficient discriminatory power to be used as standalone diagnostic tools for malignancy prediction.

In conclusion, both biochemical markers and cytopathological findings serve as critical tools for assessing malignancy risk in thyroid nodules. Conventional thyroid function tests (TSH, free T3, and free T4) did not differentiate between the benign and malignant groups in our study, but the observed association of both TSH and the TSH/T4 ratio with advancing Bethesda categories suggests potential diagnostic utility. Although elevated TSH levels consistently correlate with increased malignancy risk across studies, the prognostic significance of thyroid hormones remains indeterminate. This unresolved relationship underscores the need for rigorously controlled, large-scale investigations. Findings from such research could help clinicians use laboratory tests more effectively to predict malignancy risk and guide the management of patients with thyroid nodules.

## CONCLUSION

The TSH level and TSH/T4 ratio showed weak correlations with Bethesda categories, but no parameter demonstrated diagnostic value for malignancy prediction in ROC analysis. Therefore, these biochemical markers appear to have limited clinical utility in differentiating benign from malignant thyroid nodules. These findings suggest that thyroid function tests have limited utility in differentiating cytological malignancy, while supporting further exploration of TSH as a potential biomarker. Future studies with larger malignant cohorts are required to validate these findings and clarify any potential clinical role.

## Data Availability

Data supporting the findings of this study are available upon request from the corresponding author, İrem Şenoymak.
